# Metformin and tBHQ Treatment Combined with an Exercise Regime Prevents Osteosarcopenic Obesity in Middle-Aged Wistar Female Rats

**DOI:** 10.1155/2021/5294266

**Published:** 2021-08-14

**Authors:** Rafael Toledo-Pérez, Stefanie Paola Lopéz-Cervantes, David Hernández-Álvarez, Beatriz Mena-Montes, Gibran Pedraza-Vázquez, Carlos Sánchez-Garibay, Norma Edith López-Diazguerrero, Mina Königsberg, Armando Luna-López

**Affiliations:** ^1^Posgrado en Biología Experimental, Depto. de Ciencias de la Salud, Universidad Autónoma Metropolitana Unidad Iztapalapa, CDMX 09340, Mexico; ^2^Laboratorio de Bioenergética y Envejecimiento Celular, Depto. de Ciencias de la Salud, Universidad Autónoma Metropolitana, Unidad Iztapalapa, CDMX 09340, Mexico; ^3^Departamento de Investigación Básica, Instituto Nacional de Geriatría, CDMX 10200, Mexico; ^4^Doctorado en Ciencias Biológicas y de la Salud, Universidad Autónoma Metropolitana, CDMX, Mexico; ^5^Departamento de Neuropatología, Instituto Nacional de Neurología y Neurocirugía “Manuel Velasco Suárez”, CDMX 14269, Mexico

## Abstract

Osteosarcopenic obesity (OSO) is characterized by bone density, mass, and muscle strength loss, in conjunction with adipose tissue increase. OSO impairs physical activity and mobility, provoking autonomy loss; also, it is known that augmenting body fat in the elderly decreases life expectancy. The main factors influencing this health deterioration are the inflammatory environment induced by adipose tissue and its infiltration into muscle tissue, which leads to oxidative stress generation. Currently, there are several treatments to delay OSO, among which exercise training stands out because it improves muscle fiber quality and quantity and decreases adipose tissue. We have recently demonstrated that the combined treatment between moderate exercise and metformin slows sarcopenia's onset by a mechanism that includes adipose reduction and REDOX regulation. On the other hand, tert-butylhydroquinone (tBHQ) is a well-known antioxidant that counteracts oxidative stress. Therefore, to slow down obesity's harmful effects on muscle mass and bone mineral density, we performed different interventions, including combining a Fartlek-type exercise routine with metformin and tBHQ administration, in a model of middle-aged female Wistar rats with obesity induced with a hypercaloric diet. Our results showed that the combined exercise-metformin-tBHQ treatment increased muscle mass and strength, decreased body weight, body mass index, and fat percentage, and improved redox status, thus increasing animal survival.

## 1. Introduction

Osteosarcopenic obesity (OSO) is a phenomenon characterized by increased adipose tissue, bone mass loss (osteopenia/osteoporosis), and muscle mass loss (sarcopenia) in conjunction with strength loss (dynapenia) [1, 2].

In humans, body composition changes start at 20 years of age, mainly increasing adipose tissue by 20 to 40%, reaching up to 60% at 80 years old [1, 3]. Another change occurring during aging is muscle mass loss; 30 to 50% of muscle mass can be lost at 60 years old [1]. An epidemiological study performed in Mexico City, including 434 women over the age of 50, reported that 33.7% presented sarcopenia, 72.6% were obese, and 77.8% had osteopenia/osteoporosis, while 19% had OSO [2]. OSO development is multifactorial as it relies on poor nutrition, low physical activity, environmental factors, inflammation, and oxidative stress [4]. The latter two are promoted by the excess of adipose tissue, which is known to cause a low-grade inflammatory process by the constant release of cytokines such as tumor necrosis factor *α* (TNF-*α*), interleukin-1 (IL-1), interleukin-6 (IL-6), and C-reactive protein. Finally, the inflammatory environment leads to decreased muscle protein synthesis and increase in myofibrillar protein degradation [5].

Currently, different therapies have been used to treat OSO, both pharmacological and nonpharmacological. Some drugs like metformin and tert-butylhydroquinone (tBHQ) stand out within pharmacological therapies, while the most common nonpharmacological therapies are diet and exercise.

TBHQ has been used to prevent mice obesity undergoing a high-fat diet (HFD) because it reduces weight, decreasing adipose tissue due to the activation of acyl-coenzyme A oxidase-1 (ACOX1), an enzyme involved in fatty acid beta-oxidation. However, it has been reported that high doses of tBHQ or for prolonged time induce liver damage [6]. In *in vitro* experiments, it has been used as a hormetic treatment to protect L6 myoblasts from palmitate toxic effects [7].

Metformin (MTF) is a drug that has been used for more than 60 years to treat type II diabetes [8]; recently, its safe use and research results have positioned it as a treatment to reverse sarcopenia, since it is involved in muscle restoration and adipose tissue decrement [9–11]. In this way, a previous study from our laboratory showed that MTF improves strength and muscle mass in aged rats [12].

In regard to the nonpharmacological interventions, it has been reported that aerobic exercise improves endothelial function, increases muscle strength and mass, and remineralizes bones [13–15]. Among the different training routines, Fartlek-type exercise highlights above the rest because this aerobic training consists of changing the pace over a given time interval [16, 17]. When the pace increases, the subject is forced to reach a maximum oxygen expenditure corresponding to 70-50%, and during the breaks between each change of pace, the oxygen expenditure reaches between 40 and 30% [18]. Routines that include changes in pace are known to have higher energy expenditure in a shorter time, which results in an ideal routine for those who do not have time to perform prolonged routines [16, 18].

This type of routine is suitable for undertrained, no-trained people, or older people, as it allows them to plan and perform the routine depending on the patient's condition, evaluating parameters such as heart rate or oxygen consumption [18].

Hormesis is a process in which intermittent exposure to low doses of a harmful agent or stimulus generates a beneficial adaptive response for the cell or the organism, allowing it to survive and adapt to that stimulus [19, 20]. In biological models, a protective response has been found at low doses; however, if the dose is increased, it is possible to reach a point where stress is generated, and the response can be harmful to the cell or the organism [7, 19, 20]. An example of this effect is the response against reactive oxygen species (ROS), which at low doses can act as second messenger, while at high doses, can cause damage to biomolecules promoting cell death [19].

It has been recognized that exercise induces a hormetic mechanism [20] since it generates cellular stress by increasing the oxygen demand and ROS production. Simultaneously, a decrease in ATP levels causes the oxidation of molecules such as glucose and fatty acids to restore ATP normal levels [13]. In summary, exercise is a process that generates an energy deficit, similar to caloric restriction, which is also recognized as a hormetic mechanism [19]. This response activates an adaptive mechanism that includes fatty acid oxidation and decrease glycolytic activity, which promotes body fat loss in organisms [13]

It has been observed that in aged and malnourished rats, treatment with low concentrations of tBHQ during one week generates an antioxidant response [21, 22], while at higher concentrations, it turns out to be toxic [21]. Also, a short-time exposure activates the ACOX1 enzyme involved in the beta-oxidation of fatty acids [6].

Likewise, studies with MTF at doses of 250 mg/kg have been shown to have efficacy in muscle proliferation and differentiation [11]. In addition, recent studies using MTF for six months have shown better prevention of sarcopenic obesity compared to one-year treatment [12].

Hence, the aim of this study was to evaluate the effect of the combined intervention of Fartlek-type exercise with intermittent hormetic MTF-tBHQ treatment to prevent OSO in middle-aged female Wistar rats fed with a high-fat diet. Different studies have reported that there is a higher incidence of sarcopenic obesity in women during aging, due to the accumulation of visceral fat that occurs in postmenopause. Additionally, a decrease in bone mass density, muscle mass and strength have been observed in postmenopausal women, which explains the higher incidence of OSO compared to men [46–49]. Therefore, since the highest incidence of this disease is in women, we decided to use female rats for our study. Our results showed that the triple treatment prevented muscle mass and strength loss and maintain muscle integrity, decreased body weight, Lee's index, body fat, and improved redox status, thus increasing rat's survival.

## 2. Material and Methods

### 2.1. Animals

For this study, 84 female Wistar rats were used. The rats were provided by the UAM-I closed colony and were kept in the following conditions: four animals per polycarbonate box, an inverted cycle of light-dark (L12/D 12), under a temperature of 22 ± 2°C, water, and food *ad libitum*. All procedures with animals were strictly carried out according to the National Institutes of Health Guide for the Care and Use of Laboratory Animals, the Principles of the Mexican Official Ethics Standard 062-ZOO-1999, and the Standard for the disposal of biological waste (NOM-087-ECOL-1995).

### 2.2. Experimental Groups

At 21 days of age, the 84 rats were distributed into the Standard Diet (SD) group (*n* = 24) and the high-fat diet (HFD) group (*n* = 60). The animals were euthanized at 15 months of age. At 9 months of age, six groups were formed from the HFD rats as follows:

1. *HFD-SED*: sedentary animals fed with HDF
2. *HFD-SED + MTF*: sedentary rats fed with HFD and treated with MTF from 10 to 15 months of age
3. *HFD-SED + tBHQ*: sedentary rats fed with HFD and treated with tBHQ from 10 to 15 months of age
4. *HFD-SED + MTF + tBHQ*: sedentary rats fed with HFD and treated with MTF and tBHQ from 10 to 15 months of age
5. *HFD + EX*: rats fed with HFD along with Fartlek-type exercise from 10 to 15 months of age
6. *HFD + EX + MTF + tBHQ*: rats fed with HFD treated with MTF and tBHQ along with Fartlek-type exercise from 10 to 15 months of age

All groups included 12 females each, except for the HFD-SED + MTF (*n* = 6), HFD-SED + tBHQ (*n* = 6), and HFD + EX + MTF + tBHQ (*n* = 9), because not all animals reached 300 grams of weight (less than mean minus two standard deviations from the HFD group).

### 2.3. Animal Diets

HFD diet was prepared based on the protocols previously reported [23], based on an obesogenic diet with 23.5% protein, 20% animal lard (40% saturated fats), 5% corn oil, (60.7% polyunsaturated fats, 24.3% monounsaturated fats, 15% saturated fats, 0% cholesterol), 20.2% polysaccharides, 20.2% of simple sugars, 5% of fiber, 5% of mineral mix, and 1% of vitamins (*w*/*w*, caloric intake, 4.9 kcal/g). The SD groups were fed with Abene BDL-7100 diet containing 23% protein, 4.5% fat, and 46.5% carbohydrates (p/p, caloric intake, 3.2 kcal/g). The weekly food and water consumption was measured, and the average was plotted monthly. The results represent the consumption per box divided by the number of animals per box.

### 2.4. Metformin and tBHQ Administration

MTF and tBHQ were used as intermittent treatments to induce a hormetic response. MTF and tBHQ were orally administered at a dose of 250 mg/kg/day [10, 11] and 10 mg/kg/day [6], respectively, for 7 days per month from 10 to 15 months of age.

### 2.5. Exercise Routine

The Fartlek aerobic exercise is a continuous running exercise on the treadmill with different time and speed intervals, alternating faster and slower segments. It was performed from 10 to 15 months of age, training 5 days per week, 30 min per session. The first 10 min consisted of light exercise (25 cm/s), the next 10 min were moderate exercise (50 cm/s), and the last 10 min were again light exercise (25 cm/s). The Fartlek-type exercise routine started at 10 months of age, but at 9 months, a preconditioning training for the treadmill (Panlab/Harvard Apparatus) was performed.

### 2.6. Morphologic Parameters

Animals were weighed and measured at 3, 7, 9, and 14 months of age. The weight and nasorectal length were used to obtain the BMI and the Lee index. The BMI was determined by dividing the weight of the animal expressed in Kg by the nasorectal length expressed in meters squared (Kg/m^2^). The Lee index was obtained using the weight cubic root expressed in g, divided by the nasorectal length expressed in cm (∛g/cm). BMI is commonly used to diagnose obesity in humans, and the Lee index is used to diagnose obesity in small animals, where those with an index greater than 0.30 are considered obese animals [24].

### 2.7. Biochemical Parameters

The biochemical parameters were determined at 3, 9, and 14 months of age. The animals underwent an 8-hour fast before the studies; 200 *μ*L of blood were obtained from the tail vein of the rats; the blood was immediately analyzed to determine glucose, total cholesterol, HDL, triglycerides, creatinine, AST, ALT, and GGT, with the SPOTCHEM EZ analyzer (SP-4430) [12].

### 2.8. Body Composition (DXA)

Body composition, body fat, bone mineral density, and total lean mass without bone were determined using the Dual X-ray Absorptiometry (DXA) scanner (Discovery QDR Series, Hologic® Discovery) [12], at 3, 7, 9, and 14 months of age. The equipment was calibrated with the rat model (Hologic Rat Step Phantom P/N 010-0758). Rats were anesthetized with a cocktail containing 43% ketamine, 43% saline solution, and 14% xylazine, and 1 *μ*L/g body weight intraperitoneal (IP) injection.

### 2.9. Forelimb Grip Strength

The grip strength was evaluated 3, 7, 9, and 14 months of age, as described previously [12, 25], with some modifications. The dynamometer Rhino BAC-20 digital dynamometer (PKCh) was placed vertically and fixed to a metal grating with 1 cm apart from each metal bar. The animals were placed on the grid in a vertical position, and the tail was pulled in the same plane. The maximum tension applied just before the grip lost was recorded as the maximum grip force. These values were normalized to body weight and expressed in N/Kg. Grip strength was repeated three times for the forelimb of each rat.

### 2.10. Histology

The rats were euthanized at 15 months of age, and the gastrocnemius muscle and liver were collected to evaluate the structure and integrity using hematoxylin/eosin staining (H&E). The tissue samples were preserved in 4% neutral formalin (PBS + 4% formaldehyde) at 4°C, and subsequently, were processed in a Leica TP1020 Automatic Tissue Processor and embedded in paraffin blocks using a tissue inclusion center LEICA EG 1160. The refrigerated blocks at -20°C were cut in the transversal plane in 3 *μ*m thick sections with a LEICA RM 2125RT microtome. The slides were deparaffinized in an oven at 100°C for 15 min for their subsequent staining, through xylol, ethanol, distilled water, Harris hematoxylin, acid alcohol 5%, 5% ammoniacal water, and yellowish eosin, and to finish washing with ethanol and xylol. Synthetic resin was placed on each slide, and a coverslip was placed on top [26]. Finally, they were left to dry for 24 h for further analysis and interpretation using a Carl Zeiss Primo Star Image Analyzer with an integrated Zeiss Axiocam ERc 5S camera. Emphasis was placed on perimisium, a central region in muscle and liver parenchyma, as they are regions severely affected during OSO.

### 2.11. Redox State (GSH/GSSG Ratio)

The high-performance liquid chromatography (HPLC) method determined the content of reduced glutathione (GSH) and oxidized glutathione (GSSG) in both gastrocnemius muscle and whole blood of rats of 15 months of age following the protocol described [12] with some modifications. 50 *μ*L of blood were lysed with 200 *μ*L of lysis buffer to obtain protein, while for muscle, 200 mg of tissue were homogenized in 800 *μ*L of lysis buffer (10% hydrochloric acid/1 mM BPDS). The suspension was centrifuged at 5000 xg for 10 min at 4°C. The supernatant was recovered, and 50 *μ*L of each sample was injected into the HPLC system.

The analysis was conducted using the Waters 1525 Binary HPLC Pump (Waters, Miami, FL, USA) coupled to a 2489 UV/visible detector calibrated at 210 nm. The stationary phase was a 4.6 × 250 mm Zorbax Eclipse XDB-C18 column with 5 *μ*m of particle and as mobile phase 1% of acetonitrile and 99% of a monobasic potassium phosphate buffer (20 mM KH_2_PO_4_) at pH 2.7 was used. The flow rate from min 0 to min 5 was 1 mL/min (1% acetonitrile and 99% phosphate buffer), from min 5 to min 8 was 1.1 mL/min (10% acetonitrile and 90% phosphate buffer), and finally, from min 8 to min 10 was 1 mL/min (1% acetonitrile and 99% phosphate buffer). GSH and GSSG were detected at 210 nm using a 2489 UV/Vis detector (Waters, MA). Glutathione calibration curves were prepared using GSH and GSSG standards (5, 10, 25, 50, 100, 200, and 400 *μ*M).

### 2.12. Adiponectin Levels

Serum adiponectin levels were determined by isolating serum from rats of 15 months under the different treatments. Two *μ*L of serum were diluted 1 : 800, and 50 *μ*L of each sample were used for the analysis. The commercial kit ab108784-Adiponectin Rat ELISA Kit (Abcam) was used as described by the manufacture.

### 2.13. Statistical Analysis

All data results are presented as mean ± standard deviation of each animal group. The population growth curves were established by polynomial correlations. The differences between the groups were determined by ANOVA followed by a post hoc Holm-Sidak test, using the GraphPad prism 8.0. In all cases, the significance used is mentioned in each figure. *p* < 0.05 was considered statically significant.

## 3. Results

### 3.1. Osteosarcopenic Obesity Diagnosis

#### 3.1.1. Morphometric Parameters of SD and HFD

**Figure 1:**
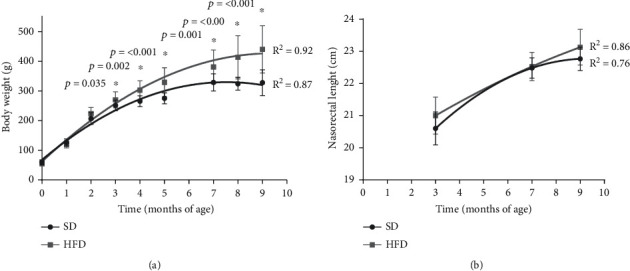
Body weight and nasorectal length of SD and HFD. Weight (a) and length (b) were evaluated in rats feed with standard diet (SD) and high-fat diet (HFD). The growth curves were established using an exponential adjustment (*R*^2^). The significant differences between groups with respect to the HFD is marked with ^∗^. The exact probability value is indicated in the graph. The comparisons were established using ANOVA and a post hoc Holm-Sidak, SD *n* = 24, HFD *n* = 60, ^∗^*p* < 0.05.

**Figure 2:**
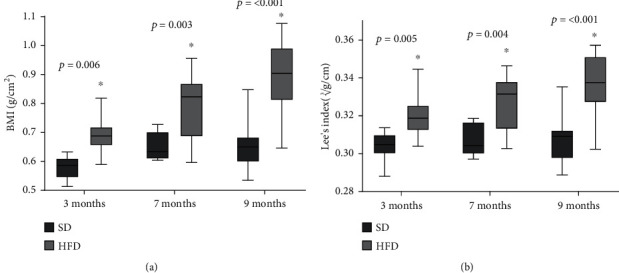
BMI and Lee's index of SD and HFD. BMI (a) and Lee's index (b) plotted as mean and standard deviation. The data were evaluated in rats feed with standard diet (SD) and high-fat diet (HFD). The significant differences between groups with respect to the HFD is marked with ^∗^. The exact probability value is indicated in the graph. The comparisons were established using ANOVA and a post hoc Holm-Sidak *n* = 10, ^∗^*p* < 0.05.

The OSO onset was determined in rats fed with the HFD vs. the ones fed with the SD before administrating the different treatments.  Figure 1(a) shows the plotted body weight determined at different time points in the rats fed with SD and HFD, starting at 21 days of birth up to 9 months of age. We found that HFD rats were heavier than the SD group from the 3rd until the 9th months of age. The data were transformed into a polynomial curve to evaluate and compare the two populations. At 3 and 4 months of age, a significant increment was found in the HFD group vs. the SD group, 7% (*p* = 0.035) and 14% (*p* = 0.002), respectively. These increases continued until the last evaluation, where the HFD group was 34% heavier than the SD group (*p* = <0.001).  Figure 1(b) shows the nasorectal length of both animal groups at 3, 7, and 9 months old. As expected, due to animal growth, the length increased according to age during 3 to 7 months, where there was a 10% growth in both groups, but no statistical differences were found, indicating that both groups increased their length as they aged, but there were no differences due to the diet. Using the body weight and nasorectal length data, the BMI was determined.  Figure 2(a) shows that at 3-month-old, HFD rats increased 20% their BMI (*p* = 0.006) with respect to the SD rats. At 7 months-old, the increment was 23% (*p* = 0.003) and at 9 months old, 37% (*p* = <0.001).  Figure 2(b) shows that Lee's index increased 2% at 3 months old (*p* = 0.005) in the HFD group, and this difference became more noticeable with time, until the 9th month of age, where the HFD group increased the Lee index in 7% compared to the SD group (*p* = <0.001). Considering Lee's index, the rats in the HFD group were considered obese from 3 months of age (0.319) to 9 months of age (0.337).

#### 3.1.2. Body Composition and Forelimb Grip Strength of SD and HFD

**Figure 3:**
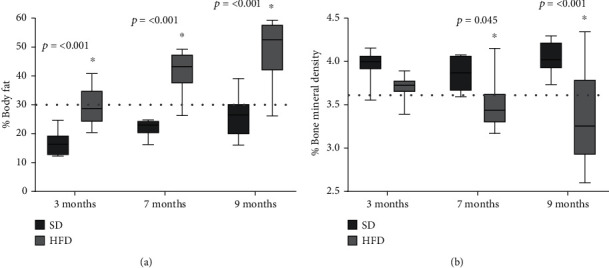
Body fat and bone mineral density of SD and HFD. Fat (a) and bone (b) percentage plotted as mean and standard deviation. The data were evaluated in rats feed with standard diet (SD) and high-fat diet (HFD) as described in the methodology section. The dotted line represents the cohort point, fat: established at 30%, bone: stablished with the mean values of young adult rat at 9 months less two and a half standard deviations. The significant differences between groups with respect to the HFD is marked with ^∗^. The exact probability value is indicated in the graph. The comparisons were established using ANOVA and a post hoc Holm-Sidak, *n* = 10, ^∗^*p* < 0.05.

The body fat evaluated by DXA is shown in Figure 3(a). At 3 months of age, the HFD group had 29% of body fat, representing 13% more than the SD group (*p* < 0.001). While at 7 months, the HFD group had 41% of body fat, representing 19% more than the SD group (*p* < 0.001), and finally, at 9 months, the HFD group had 48% of body fat, meaning 23% more fat than the SD group (*p* < 0.001). According to the cohort point set previously [27, 28], where it was established that animals with 30% or more body fat are obese animals, our results showed that the HDF group could be considered obese from 7 months of age, while the SD animals, were not obese at any time.

Regarding the bone mineral density determination (Figure 3(b)), our results showed that both groups, SD and HFD, had a similar bone percentage, 3.9 and 3.7%, respectively, and no significant difference was found at 3 months of age. At 7 months age, the HFD group had 3.4% bone, which represented 0.4% less bone than the SD group (*p* = 0.045), and at 9 months, the HFD group had 3.3% bone, meaning 0.7% less total bone mineral density compared with the SD group (*p* < 0.001). The cohort point set for osteoporosis was established as the measurement when the bone mineral density values were less than two and a half standard deviations concerning the mean value of a young adult rat with SD at 9 months of age [29]. Rats fed with SD did not present osteoporosis during the 9 months, while the HFD group showed a bone percentage lower than 3.56% from 7 months on, indicated that from that time, they had osteoporosis.

**Figure 4:**
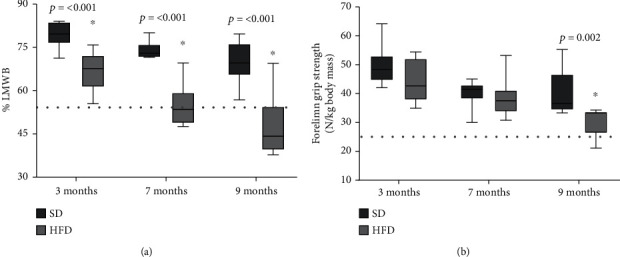
Lean mass without bone (LMWB) and forelimb grip strength of SD and HFD. LMWB (a) and grip strength (b) plotted as mean and standard deviation. The data were evaluated in rats fed with standard diet (SD) and high-fat diet (HFD) as described in the methodology. The dotted line represents the cohort point, LMWB and strength: stablished with the mean values of young adult rat at 9 months less two standard deviations. The significant statistical differences between groups with respect to the HFD is marked with ^∗^. The exact probability value is indicated in the graph. The comparisons were established using ANOVA and a post hoc Holm-Sidak, *n* = 10, ^∗^*p* < 0.05.

Figure 4(a) shows the total lean mass without bone (LMWB) determined by DXA, which represents an indirect value of the organism's muscle tissue. At 3 months old, the HFD group showed 66.6% muscle, 13% less muscle than the SD group (*p* < 0.001). At 7 months of age, the rats in the HFD group presented 54.8% LMWB, 19% less muscle than the SD group (*p* < 0.001), and finally, at 9 months, the HFD group had 47.84% LMWB, 22.8% less muscle than the SD group (*p* < 0.001). The values determined for both groups were compared against the established cohort points [30], with the muscle mass of young adult rats at 9 months with two standard deviations, and those values that were less than 55.6% muscle mass were considered as presarcopenic. The SD group did not lose muscle mass during the 9 months evaluated. Conversely, the HFD group at 7 and 9 months of age presented presarcopenia since those animals decreased 47.8% of their muscle mass. With these data, we concluded that at 7 months of age, the HFD diet generated a presarcopenia state.

The grip strength was evaluated by dynamometry (Figure 4(b)) to complete the characterization of the sarcopenic phenotype. No differences in force were found at 3 and 7 months of age between the groups, but at 9 months, the HFD group showed a 30 N forelimb grip strength (the value was normalized with animals' body weight); the value represented 35% less than the strength determined for the SD group (*p* = 0.002). When the forelimb grip strength of HFD was compared with the cohort points for force (24.7 N), none of the values was less than the cohort point.

#### 3.1.3. Food and Water Consumption of SD and HFD

Food consumption was evaluated monthly (Supplementary Figure 1a). Our determinations showed that from the first month, the SD group consumed 30% more food (measured in g) compared to the HFD group (*p* < 0.001). This same behavior was observed in the subsequent months, being the food consumption 15, 26, and 24% higher in the SD group during the 2nd, 3rd, and 4th months, respectively (*p* < 0.001). After that time, the difference between the two diets was lost. However, in Supplementary Figure 1b, we calculated the monthly kcal consumption, multiplying the kcal per gram contribution of each diet. We found that the HFD group consumed more kcal during the 9 evaluated months. In the first month, these rats consumed 14% more kcal than the SD group (*p* = 0.012), in the second month, 28% more than the SD group (*p* < 0.001), and in the third month, 17% more (*p* < 0.001). The difference was maintained until the 9th month, where the HFD group consumed 34% more kcal than the SD group (*p* < 0.001).

Water consumption was also assessed to determine the onset of metabolic syndrome (polydipsia). The results in Supplementary 1c show that the SD group consumed more water in the first months. In month 1, they consumed 46% more water (*p* = 0.003), in month 2, they consumed 26% more water (*p* = 0.008), and in month 3, the SD group consumed 17% more water than the HFD group (*p* = 0.008). After the 4th month, no statistical difference was observed.

#### 3.1.4. Biochemical Parameters of SD and HFD

Alanine aminotransferase (ALT), aspartate aminotransferase (AST), gamma-glutamyl transpeptidase (GGT), cholesterol, high-density lipoprotein (HDL), triglycerides, creatinine, and glucose were evaluated at 3 and 9 months of age. The results presented in Table 1 show that although some parameters, such as ALT and HDL, significantly decreased at 9 months and some like triglyceride increased in the HFD group, none of these values were outside the ranges reported by the clinical parameters for laboratory Wistar rats [29].

### 3.2. Hormetic Treatments to Reverse Osteosarcopenic Obesity

As a result of the HFD consumption, at 9 months of age, HDF-treated rats developed osteosarcopenic obesity. From this point on, the animals were randomly separated into 6 groups for different interventions, as described in the methodology section. Still, HFD rats that weighed less than 300 g were discarded from the study. Rats were treated from 10 to 15 months of age. Afterwards, the rats were euthanized, the tissues were stored at -80°C for further experiments. The same parameters determined with SD and HFD were evaluated in all the 6 HFD-treated groups at 14 months of age.

#### 3.2.1. Morphometric Parameters of HFD-Treated Groups

**Figure 5:**
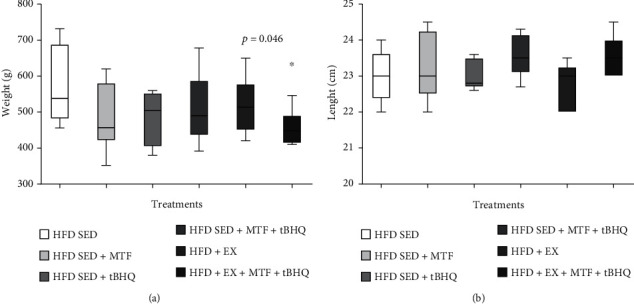
Body weight and nasorectal length of HFD-treated groups. Weight (a) and length (b) mean and standard deviation. The data were evaluated in (HFD) rats and the respective treatments at 14 months of age as described in the figure. The significant statistical differences between groups with respect to the HFD-SED are marked with ^∗^. The exact probability value is indicated in the graph. The comparisons were established using ANOVA and a post hoc Holm-Sidak. Body weight: HFD-SED *n* = 8, HFD-SED + MTF *n* = 5, HFD-SED + tBHQ *n* = 4, HFD-SED + MTF + tBHQ *n* = 11, HFD + EX *n* = 11, HFD + EX + MTF + tBHQ *n* = 8, length *n* = 5, ^∗^*p* < 0.05.

Figure 5(a) displays the bodyweight determined in the 6 HFD groups at 14 months of age. The results indicated a weight loss after the treatments, but only the group HFD + EX+ MTF + tBHQ (triple treatment) presented a significant difference against the HFD-SED group, showing a 24% reduction (*p* = 0.046). No differences in the animal's size were obtained regardless of the treatment applied (Figure 5(b)).

**Figure 6:**
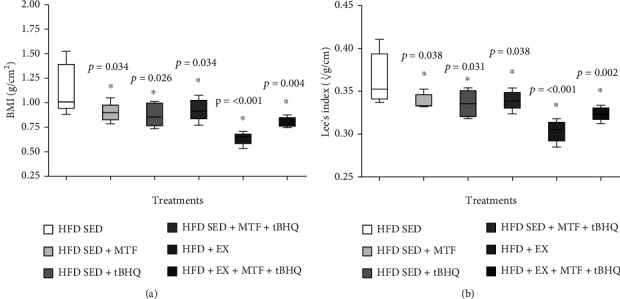
BMI and Lee's index of HFD-treated groups. BMI (a) and Lee's index (b) plotted as mean and standard deviation evaluated in HFD-rats and the respective treatments at 14 months of age. The significant statistical differences between groups with respect to the HFD are marked with ^∗^. The exact probability value is indicated in the graph. The comparisons were established using ANOVA and a post hoc Holm-Sidak *n* = 5, ∗*p* < 0.05.

Figure 6(a) shows that the BMI decreased in all treatments. The HFD-SED + MTF group decreased its BMI by 24% (*p* = 0.034), while the HFD-SED + tBHQ group decreased it by 28% (*p* = 0.026), the HFD-SED + MTF + tBHQ group (double treatment) reduced the BMI by 21% (*p* = 0.034), HFD + EX group, 76% (*p* < 0.001), and finally, the triple treatment group decreased its BMI by 41% (*p* = 0.004).  Figure 6(b) displays Lee's index values obtained for the 6 groups at 14 months of age. Similarly, to the BMI, all treatments lowered their Lee's index, the HFD-SED + MTF group decreased it by 6% (*p* = 0.038), the HFD-SED + tBHQ group by 8% (*p* = 0.031), the double treatment group by 7% (*p* = 0.038), the HFD + EX group by 20% (*p* < 0.001), and finally, the triple treatment group by 16% (*p* = 0.002). Despite Lee's index improvement, all animals were still considered obese rats since their values did not decrease below 0.30.

#### 3.2.2. Body Composition and Forelimb Grip Strength of HFD-Treated Groups

**Figure 7:**
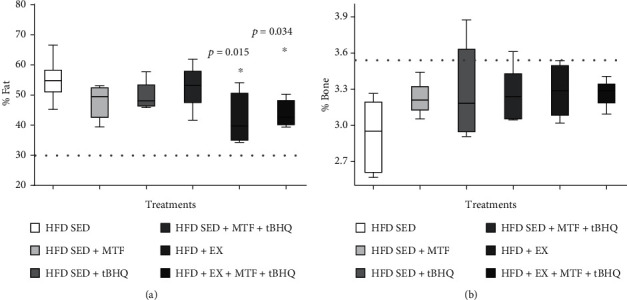
Body fat and bone mineral density of HFD-treated groups. Body fat (a) and bone mineral density (b) percent were determined in HFD rats and the respective treatments at 14 months of age. The dotted line represents the cohort point, fat: established at 30%, bone: stablished with the mean values of young adult rat at 9 months less two and a half standard deviations. The significant differences between groups with respect to the HFD are marked with ^∗^. The exact probability value is indicated in the graph. The comparisons were established using ANOVA and a post hoc Holm-Sidak *n* = 5*p* < 0.05.

The body composition was analyzed in the 6 HFD groups at 14 months of age. The fat percentage in HFD rats of different treatments was determined in Figure 7(a). The results showed that only the groups that included exercise training significantly decreased their fat percentage. The HFD + EX group showed a 42.2% fat, representing a 12% reduction with respect to the HFD-SED group (*p* = 0.015), while the triple treatment (HFD + EX +MTF + tBHQ) presented 43.8%, implying an 11% decrease (*p* = 0.015). In the significant fat reduction, no treatment managed to revert obesity since they did not lower their fat percentage below the 30% established by the cohort point of fat [27, 28]. Regarding the bone content, the results in Figure 7(b) show a slight increase in bone after the treatments, but no significant differences were observed, thus maintaining the HFD-treated groups in an osteoporotic state.

**Figure 8:**
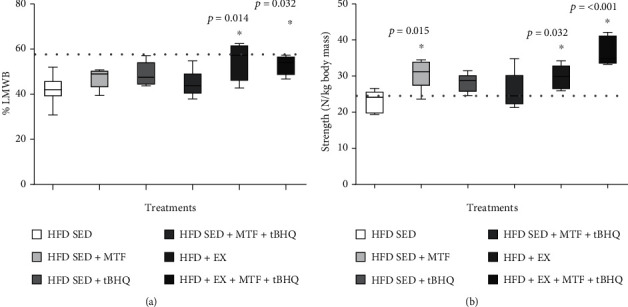
LMWB and grip strength of HFD-treated groups. LMWB (a) and grip strength (b) were determined in HFD-rats and the respective treatments at 14 months of age. The dotted line represents the cohort point [30], LMWB and strength: stablished with the mean values of young adult rat at 9 months less two standard deviations. The significant differences between groups with respect to the HFD are marked with ^∗^. The exact probability value is indicated in the graph. The comparisons were established using ANOVA and a post hoc Holm-Sidak *n* = 5. ^∗^*p* < 0.05.

Figure 8(a) shows that only the treatments that included exercise manage to restore the muscle tissue evaluated as LMWB. The HFD + EX group displayed 54.2% muscle, which represented a 12% increase compared to the HFD-SED group (*p* = 0.014), the triple treatment (HFD + EX + MTF + tBHQ) showed 52.9%, increasing 10% compared to HFD-SED (*p* = 0.014). When comparing the muscular gain with ones at 9 months (55.6%), the treatments that include an exercise routine demonstrated to restore the muscle mass to a presarcopenia point. The grip strength was evaluated to confirm this finding.  Figure 8(b) shows that MTF treatment increased the force 7% with respect to the HFD-SED group (*p* = 0.015), while the treatments that included exercise training, HFD + EX, and the triple treatment increased the strength by 9% (*p* = 0.032) and 13% (*p* < 0.001), respectively. Similarly, the strength values were compared with the dynapenia cohort point (24.7 N), and the HFD-SED group, along with all the treatments, presented a higher force than the dynapenia cohort [30], except the HFD-SED without treatment at 14 months. The finding suggests that all treatments recovered the grip strength.

#### 3.2.3. Muscle and Liver Histology of HFD-Treated Groups

**Figure 9:**
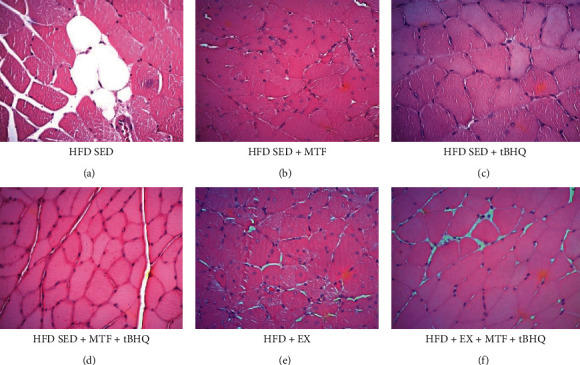
Gastrocnemius histology of HFD-treated groups. Crosssectional views of gastrocnemius stained with H&E are shown to differentiate structure and arrangement of muscle fibers obtained for the HFD rats after the different interventions at 15 months of age. Micrographs are 40x magnification. *n* = 3.

Figure 9 shows rat's gastrocnemius sections at 15 months of age stained with H&E.  Figure 9(a) displays the HFD-SED group gastrocnemius. Fascicles derangement is observed, evidencing the muscle fiber separation and a greater amount of endomysium and intramuscular adipose tissue. The adipocytes of medium size (approximately 25 *μ*m) are observed along with inflammatory infiltrates between the muscle fibers. In the case of the HFD-SED + MTF (Figure 9(b)), HFD-SED + tBHQ (Figure 9(c)), and double treatment (HDF-SED + MTF + tBHQ) (Figure 9(d)) groups, the muscle fibers show a better arrangement, the fascicles are better defined, and the spaces between muscle fibers are smaller, although the infiltrates can be still observed between the fascicles. In regard to the groups that performed the exercise routine (HFD + EX) (Figure 9(e)) and the triple treatment (HFD + EX + MTF + tBHQ) (Figure 9(f)), a closer union between the muscle fibers is observed. Moreover, one fascicle can hardly be distinguished from another; however, they still present inflammatory infiltrates.

**Figure 10:**
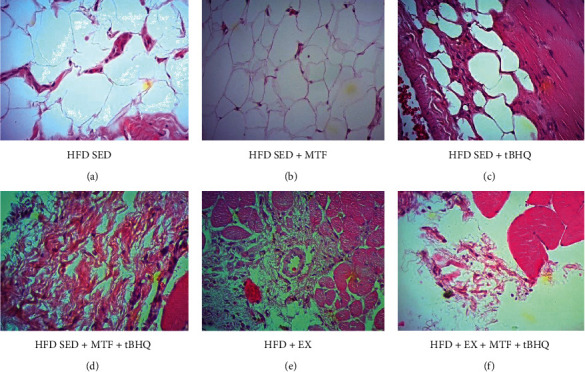
Gastrocnemii's epimysium histology of HFD-treated groups. Representative images that show crosssections of the gastrocnemii's epimysium stained with H&E to differentiate the cell type and the tissue arrangement. The gastrocnemius was obtained for the HFD rats after the different interventions at 15 months of age. Micrographs are 40x magnification. *n* = 3.

Figure 10 shows the gastrocnemius epimysium, because in this region, different cell types and different connective tissue patterns were observed. In the HFD-SED group (Figure 10(a)), numerous large adipocytes (approximately 50 *μ*m) were observed in a polyhedral form, as well as dense connective tissues located in the periphery of the fascicles. In the HFD-SED +MTF group (Figure 10(b)), there was a reduction in the adipocytes size and number, although dense connective tissues are still appreciated. This same feature was observed in the double treatment group (HFD-SED +MTF + tBHQ) (Figure 10(d)), and in the HFD-SED + tBHQ-treated group (Figure 10(c)), where there was a decrease in adipocytes size (about 30 *μ*m) and a greater proportion of dense connective tissues among them compared to the group without treatment. Finally, in the exercise groups (HFD + EX) (Figure 10(e)) and the triple treatment (HFD + EX+ MTF + tBHQ) (Figure 10(f)), a lower amount of adipocytes was observed. However, it is important to notice that the exercise group (HFD + EX) showed a higher proportion of dense connective tissues infiltrated between the muscular fascicles. It was observed in the entire epimysium periphery, while the triple treatment group decreased its dense connective tissue, and in some regions, loose connective tissue was observed.

**Figure 11:**
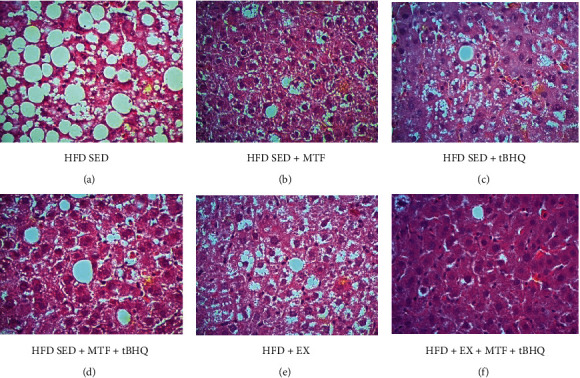
Liver histology of HFD-treated groups. Crosssections of liver stained with H&E are shown to differentiate structure and infiltrates. The gastrocnemius was obtained for the HFD rats after the different interventions at 15 months of age. Micrographs are 40x magnification. *n* = 3.

Since one of the most important organs for lipid metabolism is the liver, we decided to analyze its architecture. In the HFD-SED group (Figure 11(a)), lipid drops of various sizes (from 10 to 30 *μ*m) were found in more than 70% of the organ. The columnar hepatocytes architecture was lost, and in some cases, it was not possible to distinguish the central lobular vein; monocytes and lymphocytes inflammatory infiltrates were observed in large part of the organ, suggesting nonalcoholic hepatic steatosis. In the HFD-SED + MTF-treated group (Figure 11(b)), the HFD-SED + tBHQ group (Figure 11(c)), the HFD-SED + MTF + tBHQ group (Figure 11(d)), and the exercise group (Figure 11(e)), a decrease in the number of lipid drops was observed since only 20 to 30% proportion of drops were observed. Inflammatory infiltrates and some signs of hypoxia were also there, and the hepatocytes recovered their columnar shape compared to the HFD-SED group. Finally, the triple treatment (Figure 11(f)) showed a greater decrease in the proportion of lipid drops, presenting one to three lipid drops per slice. The same proportion of inflammatory infiltrate was observed in all slices.

#### 3.2.4. Food and Water Consumption of HFD-Treated Groups

With the treatments, we expected a decrease in food consumption while maintaining water consumption at 14 months of age. The results in Supplementary Figure 2a show a decrease in food consumption in the HFD-SED + MTF + tBHQ group (58%, *p* = 0.002), HFD + EX group, 68% (*p* < 0.001), and the triple treatment (65%, *p* < 0.001), compared to the HFD-SED group. The HFD-SED +MTF and HFD-SED + tBHQ treatment groups were not included in the statistical analysis since the consumptions were averaged by animal per box, and there were not enough boxes available for this analysis. In conclusion, we observed a reduction in food consumption at 14 months of age of HDF-treated groups, especially the treatments including the exercise regimen and the double treatment. When the food consumption was expressed in kcal (Supplementary Figure 2b), the same behavior was observed. The double treatment decreased kcal consumption by 50% (*p* = 0.038), the HFD + EX group by 59% (*p* = 0.027), and the triple treatment group by 47% (*p* = 0.038). Because the food values were used to determine consumption in kcal, the statistic was not achieved for the MTF- and tBHQ-treated groups, where not enough data is available for this analysis. The results indicate that the kcal consumption decreased during the combined MTF + tBHQ treatment and the treatments, including exercise. Water consumption is shown in Supplementary Figure 2c. No significant differences were found in any treatments at 14 months of age.

#### 3.2.5. Biochemical Parameters of HFD-Treated Groups

The biochemical parameters described before were determined at 14 months old in HFD animals after the different treatments. The results in Table 2 show no significant differences in the biochemical parameters compared to the HFD-SED group or compared to the laboratory-established ranges of Wistar rat clinical parameters [29]. With these results, we assure that the animals fed with HFD presented metabolic alteration at no time since none of them exceeded the parameters of the reported range.

#### 3.2.6. Animal Survival (Kaplan-Meier's Curves)

**Figure 12:**
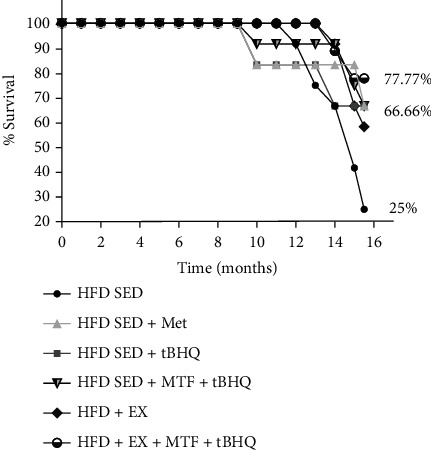
Kaplan Meier's curve of HFD-treated groups. Percent of rat's survival determined in HFD-rats and the respective treatments. Initial animal number = 81.

The Kaplan-Meier's curve was used to determine the organism's survival.  Figure 12 was performed with the data obtained during the 15 months of the study. The HFD-SED group had a lower survival rate (25%), while the exercised group (HFD + EX) had 58.3% survival, representing 33.3% more survival than HFD-SED. On the other hand, the HFD-SED + MTF, the HFD-SED + tBHQ, and the double treatment (HFD-SED + MTF+ tBHQ) groups showed 66.6% survival being 41.6% higher than the HFD-SED group. The triple treatment group presented the highest survival percentage, 77.7%, being 52.7% higher than the HFD-SED group. These results show that all treatments improved animal survival, but the group with the best result was the triple treatment which tripled the HFD-SED survival. Finally, the animals' cause of death is described below: 6% of the animals died from complications with the anesthesia; 8% were sacrificed due to cachexia; 43% were sacrificed for having tumors in the mammary glands; and for the remaining 43%, the cause of death was not determined. Within the animals that presented tumors in the mammary glands, 62.5% belonged to the HDF group and 37.5% to the HFD groups with exercise or some treatment.

#### 3.2.7. Redox State of HFD-Treated Groups

**Figure 13:**
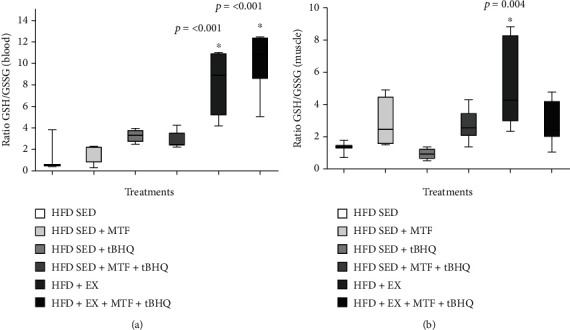
GSH/GSSG ratio of HFD-treated groups. The ratio of reduced glutathione (GSH) and oxidized glutathione (GSSG) were determined by HPLC using the gastrocnemius muscle (a) and the total blood (b) obtained from HFD-rats and the respective treatments at 15 months of age. The significant statistical differences between groups with respect to the HFD are marked with ^∗^. The exact probability value is indicated in the graph. The comparisons were established using ANOVA and a post hoc Holm-Sidak HFD *n* = 3, HFD + MTF *n* = 4, HFD + tBHQ *n* = 4, HFD + MTF + tBHQ *n* = 8, HFD + EX *n* = 7, HFD + EX + MTF + tBHQ *n* = 7. ^∗^*p* < 0.05.

Since it is known, in independent studies, that treatments with MTF, tBHQ, and exercise decreases oxidative stress with beneficial effects, we evaluated the muscle and systemic (blood) redox state at 15 months old in HFD-treated groups.  Figure 13(a) shows the gastrocnemius muscle GSH/GSSG ratio normalized with mg of protein. The HFD-SED + MTF and HFD-SED + tBHQ groups and the double treatment group did not modify their GSH/GSSG ratio. However, the groups that included exercise training improved their redox state, five times for the ones with exercise alone (HFD+EX) (*p* < 0.001) and 6 times for the triple treatment (*p* < 0.001). Our results showed that treatments that included exercise improved redox state compared to the HFD group. The GSH/GSSG ratio in total blood was also evaluated (Figure 12(b)); in this case, the only significant difference was found in the HFD + EX group, having 4 times increase compared to the HFD group (*p* = 0.004). We confirmed that the exercise regimen improved the redox state in both muscle and total blood with these results.

#### 3.2.8. Serum Adiponectin Levels in HFD-Treated Groups

**Figure 14:**
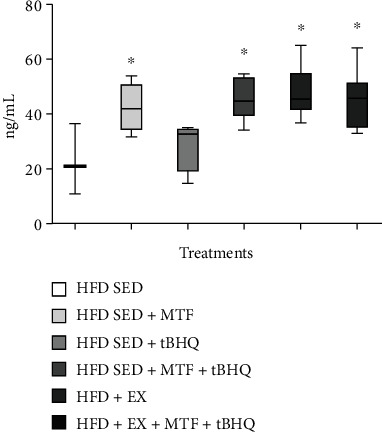
Adiponectin levels in HFD-treated groups. Adiponectin serum levels in HFD rats and the respective treatments at 15 months of age were determined as described in materials and methods. The significant statistical differences among groups with respect to the HFD are marked with ^∗^. The exact probability value is indicated in the graph. The comparisons were established using ANOVA and a post hoc Holm-Sidak HFD *n* = 3, HFD + MTF *n* = 4, HFD + tBHQ *n* = 4, HFD + MTF + tBHQ *n* = 8, HFD + EX *n* = 7, HFD + EX + MTF + tBHQ *n* = 7. ^∗^*p* < 0.05.

The results obtained after evaluating the adiponectin levels in the 15-month-old rat's serum after the different treatments are shown in Figure 14. The SED-HFD group presented levels of 22.7 ng/mL of adiponectin. The MTF-treated animals (HFD-SED + MTF) significantly (*p* = 0.025) increased their adiponectin levels 42.23 ng/mL, representing 1.85 times more than the HFD-SED group. In the case of the tBHQ group (HFD-SED + tBHQ), no statistical differences were found with respect to the HFD-SED. While the double treatment (HFD-SED + MTF + tBHQ) also significantly increased the adiponectin levels (*p* = 0.005) to 45.47 ng/mL, representing twice as much as the HFD-SED group. On the other hand, similar results were obtained when we analyzed adiponectin levels of the exercised groups. Likewise to the MTF-treated animals, the HFD + EX also increases the adipocytokine levels to 47.62 ng/mL, which represents 2.09 times the levels of the group without treatment (*p* = 0.004). Similar levels were determined in the triple treatment group (HFD + EX + MTF +tBHQ): 46.27 ng/mL, representing 2.08 times levels in the HFD SED group (*p* = 0.005).

## 4. Discussion

Osteosarcopenic obesity (OSO) is a phenomenon that was recognized as a combined pathology a few years ago [2], and since then, its study has taken-on great importance. It is known that OSO affects around 32% [1] of the aged population; thus, OSO is more severe than each of the individual pathologies that comprise it [1, 2]. In 2017 in Mexico, it was reported that 19% of aged Mexican females have OSO. [2]. Globally, the prevalence of OSO in woman is 11% [31]. Multiple animal models have been used to study OSO, regarding different diets, sedentary lifestyle [6, 10], and even hormonal restriction [32]. However, almost all of them were performed in young animal models, and very few had used intervention therapies to reverse or delay the disease. Hence, an OSO model has been established here using female Wister rats fed with an HFD diet. The HFD promoted fat gain and muscle loss from 3 months on, bone loss from 7 months on, and strength loss from 9 months on, thus confirming the OSO phenotype.

It is worth mentioning that there are few obesity studies in aged animals, in particular using long-term HFD supplementation. In our study, HFD was given starting after weaning and until the end of the experiment at 15 months of age when the rats were considered middle age. The different interventions carried out began at 10 months of age since the OSO was established at 9 months. The treatments included a change in physical habits (Fartlek-type exercise) and tBHQ and/or MTF administration for 5 months, which allowed the animals to be longitudinally evaluated during and after the treatments. Interestingly, the pharmacological interventions were administered only 7 days/month from 10 to 15 months of age to obtain a hormetic effect.

The weight determined for the HFD-animals at 4 months (300 g) correlates with the results reported by other authors [23, 33], indicating that our animals were kept in excellent conditions to generate the obesity model. The case of the SD-animals, which weighted 250 g, also concurred with those described by the Harlan technical reports for laboratory animals [34], demonstrating that the SD diet used meets the criteria established for normal laboratory rats.

Among the interventions used to prevent or reverse OSO, the change in diet and regular exercise stand out [35]. However, modifying the diet is known to be a challenge, because in many cases, it does not generate adherence in the patient, and they tend to return to their previous diet [36]. Therefore, the exercise routine has become a more affordable intervention. The type of training to prevent OSO has also been studied, whether using the combination of aerobic exercises, and strength exercises, high-intensity aerobic exercises, and aerobics with changes in pace (HIIT and Fartlek). The latter two have shown better results since they combine rhythm changes promoting a continuous state of stress, which impacts on heart rate that is maintained several minutes after slowing down [13, 15, 37].

Although there are reports that MTF and tBHQ individually decrease the organism's weight [6, 10], this did not happen in our study; perhaps this effect is not observed in old organisms, unlike young organisms. Only animals subjected to the triple treatment lost weight. Interestingly, the rats subjected to the interventions that included exercise decreased their fat percentage at 14 months of age, even though all treatments are known to induce fat loss [6, 10, 13] mainly by AMPK activation, which depends on the AMP/ATP ratio. MTF is known to inhibit electron transport chain complex 1 in a dose-dependent manner [38], thus decreasing ATP production and activating AMPK. Li and coworkers (2019) reported that tBHQ directly activates AMPK, although the exact mechanism is still unclear, while exercise is also known to increase AMPK activity by promoting molecular catabolism of glucose and fatty acids [13]. All the treatments are directly involved with lipid catabolism by activating beta oxidation-enzymes, as is the case of tBHQ that has been implicated in ACOX1 activation [6, 13, 37]. Moreover, exercise is the most studied mechanism related to lipid degradation since exercise promotes lipid catabolism due to the activation of various enzymes such as mLPL, a lipase known to increase its activity by 80% when people undergo moderate exercise regimen [39]. On the other hand, exercise is also involved in lipid transport within the cell; the CD36 concentrations are known to increase, and several proteins associated with this process, such as fatty acid binding proteins (FABPs), which internalize fatty acids. Once inside the cell, the fatty acids are activated by Acil-CoA synthesis (ACS), allowing their use in metabolism or storage. Furthermore, exercise has also been linked to increasing carnitine synthesis (CPT1), allowing fatty acids to enter the mitochondria, where beta-oxidation is carried out [13].

In the case of muscle mass and grip strength, the treatments that included exercise managed to recover muscle mass by at least 10%, even if the animals did not reach a percentage of muscle mass not to be considered sarcopenic. Despite being reported that MTF activates muscle cell proliferation and differentiation [10, 11, 13], the hormetic treatment with MTF failed to induce a statistical difference in muscle mass gain. Again, all the former reports were done in young animals where the muscular proliferation rate was high. When analyzing gripping force, it can be observed that the exercise treatments significantly improved animal's strength, up to a point where they were no longer considered dynapenic. These results concur with a previous study [12], where old animals regained strength but not muscle mass after the MTF and exercise treatments. Interestingly, the short-time MTF treatment (6 vs. 12 months) obtained greater benefits.

A significant finding was the animal's survival in each of the six intervention groups during the HFD. Remarkably, the group with a higher survival rate was the triple treatment. This effect might be associated with the loss of body fat and decrease of inflammation, which has already been reported as a determining factor in organism longevity since it has been suggested that the loss of body fat brings an increase in life expectancy in adults with obesity. [40]. Interestingly, animals subjected to HFD diet developed tumors in the mammary glands, so they had to be euthanized. Tumor development was 1.66 times higher in sedentary HFD-fed animals compared to the HFD animals that exercised or were subjected one of the treatments. This is consistent with some reports in which it has been determined that obesity is a significant risk factor for various cancers, including breast cancer, resulting in an increased risk, as well as morbidity and mortality. Higher inflammatory cytokine levels during obesity induce the proliferative pathways, such as macrophages infiltration, angiogenesis, and antiapoptotic pathways [50, 51]. Adipose tissue generates a chronic low-grade inflammatory state which also contributes to increased oxidative stress [8, 12]; it is known that both exercise and MTF reduce adipose tissue and consequently decrease inflammatory state and oxidative stress, and the latter have linked it to an increase in life expectancy [13, 14, 41]. Regarding the beneficial effects generated by MTF treatment, it was reported that in mice subjected to HFD and MTF, there was a weight decrease compared to mice without treatment; MTF also decreased glucose and insulin blood levels, transaminases (ALT and AST), as well as adipose tissue infiltration to organs such as the liver and pancreas. However, in that study, MTF failed to modify total cholesterol and triglycerides levels [10].

Although in studies performed in elderly patients, it was reported that MTF regulated cholesterol and LDL levels and weight loss, whether the patients presented diabetes or not, MTF was recommended for patients with overweight and people at risk from diabetes [9].

On the other hand, tBHQ has shown to be a molecule that prevents damage to tissues and cells against toxic agents in the brain [21], in the liver [42], and recently in muscle precursor cells [7], by promoting the antioxidant defense activation via the transcription factor Nrf2 [21]. Here, Nrf2 activation was not evaluated, but a change in the redox state was observed, specifically at the GSH level, so Nrf2 might suggest that this transcription factor might be participating in the beneficial outcome.

Adiponectin is an adipocytokine responsible for promoting fatty acid beta oxidation and glucose metabolism [43]. It is secreted by adipocytes and some other cell types, such as cardiac and muscle myocytes [44]. Adiponectin has also been linked to the anti-inflammatory response as it decreases TNF-*α* levels, IL-6, and NF-*κ*B as well as promoting the synthesis of anti-inflammatory cytokines such as IL-10 [45]. Our results showed that all treatments, except for tBHQ, increased adiponectin levels. This is very interesting because high levels of adiponectin are related to an increase in fatty acid oxidation and a decrease in the inflammatory state. Being the loss of body fat a constant variable in all the treatments that included an exercise regimen. This agrees with other studies where adiponectin supplementation reduced the proinflammatory profile and also the body weight, by improving glucose metabolism and beta oxidation [44].

Regular exercise training plays a vital role in maintaining good health and fitness. In exercised muscles, metabolic energy is known to increase due to high glucose utilization [13]. Fartlek-type exercise is generally studied in young organisms and athletes with previous training. However, it turned out to be a suitable alternative to prevent OSO in middle-aged female Wistar rats. This kind of training combines resistance exercise but simultaneously has a period of greater energy demand, generating more significant energy expenditure without becoming a high-intensity training and allowing old rats to complete the routine. Hence, it might also be a suitable routine for older people. Another known effect of exercise is to improve insulin sensitivity, reducing body fat, and increase muscle mass. The latter brings with it an increase in glucose membrane transporters (Glut 4), improving glucose uptake in the muscle and decreasing free blood glucose levels [13]. Our results showed that exercise is essential for muscle preservation and fat loss when HFD generated obesity. This intervention prevented and even recovered muscle mass since the HFD-SED animals at 9 months of age had 47.8% muscle, after 5 months of being subjected to the triple treatment (at 14 months of age), and increased their muscle mass to 52.9%. The rats that only performed exercise (HFD-EX) increased their muscle mass to 54.2% but survived 10% less than the triple treatment group. Although they were not as successful, the treatments where only MTF and tBHQ were used also proved to be suitable interventions to improve the adverse effects of sedentarism and hypercaloric diet.

As mentioned before, the body fat loss might be related to the activation of fatty acid catabolic pathways and AMPK activation. AMPK was not evaluated here. However, it has been reported that all the treatments activate AMPK, promoting lipid and glucose catabolism. Consequently, the body fat loss might be related to an increase in life expectancy, being the triple treatment that decreases the highest amount of body fat and increased life expectancy to a greater extent. The reduction in food consumption may also be involved in the body fat loss, and as mentioned above, the exercise routine and the MTF and tBHQ treatment reduced food consumption, generating a process similar to caloric restriction, where the cells are known to the energy stored in the triglycerides or glycogen. This process could also be synergistically contributing to body fat loss.

## 5. Conclusions

After 9 months of HDF intake, the rats presented OSO, thus becoming a suitable model for studying this disease in middle-aged female rats. The MTF treatment increased the strength and the survival rate in the OSO model, while the Fartlek-type exercise and the triple treatment increased muscle mass and strength and improved the redox state and the survival rate while decreasing the BMI, Lee's index, the body fat, and the inflammatory profile. Our results suggest that the Fartlek routine and the triple treatment are successful interventions to prevent OSO onset during rat middle age, mainly because of their effect as anti-inflammatory, metabolic regulators, and antioxidant agents. In this sense, the exercise routine is a very important factor within the triple treatment, since it proved to be effective on its own, as well as part of the triple treatment. Therefore, it could be interesting to test these interventions for a longer time, since the aged organisms do not respond in the same way as the young and the middle-age ones, and a greater difference between treatments could be found. In addition, it would be interesting to perform this kind interventions in humans, since the Fartlek-exercise routine may be safe for the elderly population, and both drugs used here are already in use.

## Figures and Tables

**Table 1:** Biochemical parameters of SD and HFD.

	Groups				*p*
	SD 3 M	HFD 3 M	SD 9 M	HFD 9 M	
AST (U/L)	30.20 ± 8.58	36.6 ± 5.03	41.8 ± 3.49	50.6.0 ± 9.76	
ALT (U/L)	11.8 ± 2.49	11.0 ± 1.73	20.4 ± 3.71	13.6 ± 4.5 **A**	*A* = 0.009
GGT (U/L)	12.2 ± 1.78	11.0 ± 1.73	14.0 ± 4.06	11 ± 1.41	
Cholesterol	60.2 ± 11.9	50.6 ± 1.51	73.8 ± 8.22	58.8 ± 12.52	
HDL mg/dL	18.0 ± 5	10.2 ± 0.83 **A**	23.8 ± 4.49	13.4 ± 4.21 **B**	*A* = 0.007 *B* = 0.001
Tryglicerides mg/dL	51 ± 6.63	35.2 ± 15.5	46.8 ± 8.25	92.2 ± 17.94 **A**	*A* = <0.001
Creatinine mg/dL	0.28 ± 0.04	0.38 ± 0.16	0.28 ± 0.10	0.28 ± 0.13	
Glucose mg/dL	107.8 ± 8.31	104.8 ± 13.29	94.4 ± 10.33	102 ± 10.44	

The table shows the mean and standard deviation of GOT, GPT, GGT, cholesterol, HDL, triglycerides, creatinine, and glucose determined in rats fed with standard diet (SD) and high-fat diet (HFD). The significant statistical differences between groups with respect to the HFD is marked with ^∗^. The exact probability value is indicated. The comparisons were established using ANOVA and a post hoc Holm-Sidak *n* = 10, ^∗^*p* < 0.05.

**Table 2:** Biochemical parameters of HFD-treated groups.

	Treated groups (14 months of age)					
	HFD SED	HFD SED + MTF	HFD SED + tBHQ	HFD SED + MTF + tBHQ	HFD + EX	HFD + EX + MTF + tBHQ
AST (U/L)	49.4 ± 8.90	58.80 ± 18.83	66.60 ± 32.12	63.40 ± 22.63	54.6 ± 18.58	58.6 ± 24.24
ALT (U/L)	15.20 ± 5.26	13.80 ± 4.49	13.80 ± 5.02	11.80 ± 3.03	10.6 ± 1.34	14.6 ± 7.40
GGT (U/L)	29.60 ± 16.47	17.80 ± 12.03	33.20 ± 36.26	13.00 ± 3.11	10.4 ± 0.89	24.2 ± 23.84
Cholesterol	60.2 ± 22.21	74.00 ± 9.30	78.20 ± 8.92	63.4 ± 11.33	63.0 ± 5.74	62.4 ± 12.68
HDL mg/dL	14.0 ± 6.51	15.00 ± 4.69	14.20 ± 5.26	14.20 ± 2.86	14.2 ± 1.28	13.6 ± 4.93
Tryglicerides mg/dL	45.45 ± 21.62	48.40 ± 28.89	69.80 ± 12.26	66 ± 30.46	49.6 ± 27.58	42.6 ± 16.56
Creatinine mg/dL	0.7 ± 0.31	0.32 ± 0.16	0.32 ± 0.21	0.48 ± 0.51	0.26 ± 0.08	0.36 ± 0.15
Glucose mg/dL	101.8 ± 7.82	95.20 ± 22.1	85.20 ± 18.16	106.6 ± 6.46	93.2 ± 13.72	106.2 ± 6.68

The table shows the mean and standard deviation of ALT, AST, GGT, cholesterol, HDL, triglycerides, creatinine, and glucose that were determined in HFD-rats and the respective treatments at 14 months of age. The significant statistical differences between groups with respect to the HFD are marked with ^∗^. The exact probability value is indicated in the graph. The comparisons were established using ANOVA and a post hoc Holm-Sidak *n* = 5. ^∗^*p* < 0.05.

## Data Availability

The data used to support the findings of this study are included within the article.

## Supplementary Materials

Supplementary 1 Supplementary Figure 1: food and water consumption of SD and HFD. Food consumption expressed in g (a) or kcal (b) and water consumption (c) plotted as mean and standard deviation were evaluated in rats feed with standard diet (SD) and high-fat diet (HFD). The growth curves were established using an exponential adjustment (*R*^2^). The significant statistical differences between groups with respect to the HFD is marked with ^∗^. The exact probability value is indicated in the graph. The comparisons were established using ANOVA and a post hoc Holm-Sidak SD *n* = 24, HFD *n* = 60, ^∗^*p* < 0.05.

Supplementary 2 Supplementary Figure 2: Food and water consumption of HFD-treated groups. Food consumption expressed in g (a) or kcal (b) and water consumption (c) were determined in HFD-rats and the respective treatments metformin (MTF), tBHQ, and exercise (EX), and their combinations. The significant statistical differences between groups with respect to the HFD were marked with ^∗^. The exact probability value is indicated in the graph (The comparisons were established using ANOVA and a post hoc Holm-Sidak HFD *n* = 8, HFD + MTF *n* = 5, HFD + tBHQ *n* = 4, HFD + MTF + tBHQ *n* = 11, HFD + EX *n* = 11, HFD + EX + MTF + tBHQ *n* = 8. ^∗^*p* < 0.05).
