# Supplementary material for: Metformin and tBHQ Treatment Combined with an Exercise Regime Prevents Osteosarcopenic Obesity in Middle-Aged Wistar Female Rats
Source: Oxid Med Cell Longev. 2021 Aug 14;2021:5294266. doi: 10.1155/2021/5294266 (PMC8383718; doi:10.1155/2021/5294266)
Supplement: Supplementary 1 — Supplementary Figure 1: food and water consumption of SD and HFD. Food consumption expressed in g (a) or kcal (b) and water consumption (c) plotted as mean and standard deviation were evaluated in rats feed with standard diet (SD) and high-fat diet (HFD). The growth curves were established using an exponential adjustment (R2). The significant statistical differences between groups with respect to the HFD is marked with ∗. The exact probability value is indicated in the graph. The comparisons were established using ANOVA and a post hoc Holm-Sidak SD n = 24, HFD n = 60, ∗p < 0.05. [file 5294266.f1.docx]

**
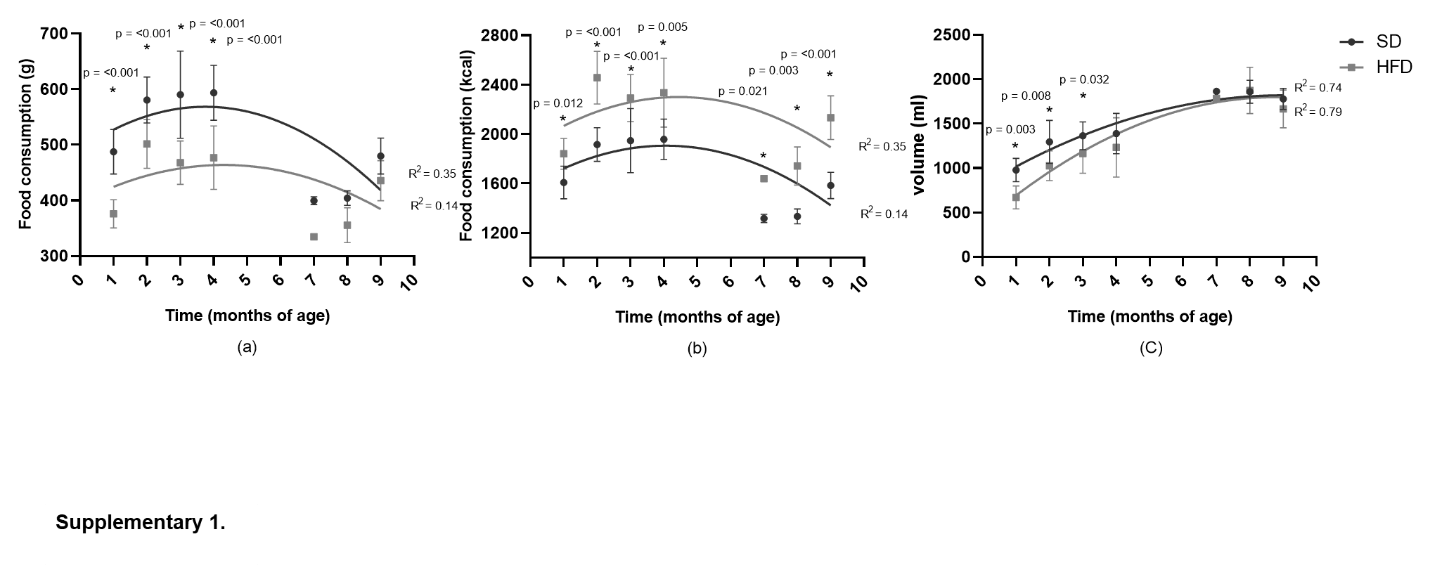
 Supplementary figure 1. Food and water consumption of SD and HFD.**

Food consumption expressed in g (**a**) or kcal (**b**), and water consumption (**c**), plotted as mean and standard deviation were evaluated in rats feed with standard diet (SD) and high fat diet (HFD). The growth curves were established using an exponential adjustment (R2). The significant statistical differences between groups with respect to the HFD is marked with *. The exact probability value is indicated in the graph. The comparisons were established using ANOVA and a post hoc Holm-Sidak SD n = 24, HFD n = 60, *p < 0.05.
