# Supplementary material for: Metformin and tBHQ Treatment Combined with an Exercise Regime Prevents Osteosarcopenic Obesity in Middle-Aged Wistar Female Rats
Source: Oxid Med Cell Longev. 2021 Aug 14;2021:5294266. doi: 10.1155/2021/5294266 (PMC8383718; doi:10.1155/2021/5294266)
Supplement: Supplementary 2 — Supplementary Figure 2: Food and water consumption of HFD-treated groups. Food consumption expressed in g (a) or kcal (b) and water consumption (c) were determined in HFD-rats and the respective treatments metformin (MTF), tBHQ, and exercise (EX), and their combinations. The significant statistical differences between groups with respect to the HFD were marked with ∗. The exact probability value is indicated in the graph (The comparisons were established using ANOVA and a post hoc Holm-Sidak HFD n = 8, HFD + MTF n = 5, HFD + tBHQ n = 4, HFD + MTF + tBHQ n = 11, HFD + EX n = 11, HFD + EX + MTF + tBHQ n = 8. ∗p < 0.05). [file 5294266.f2.docx]

**
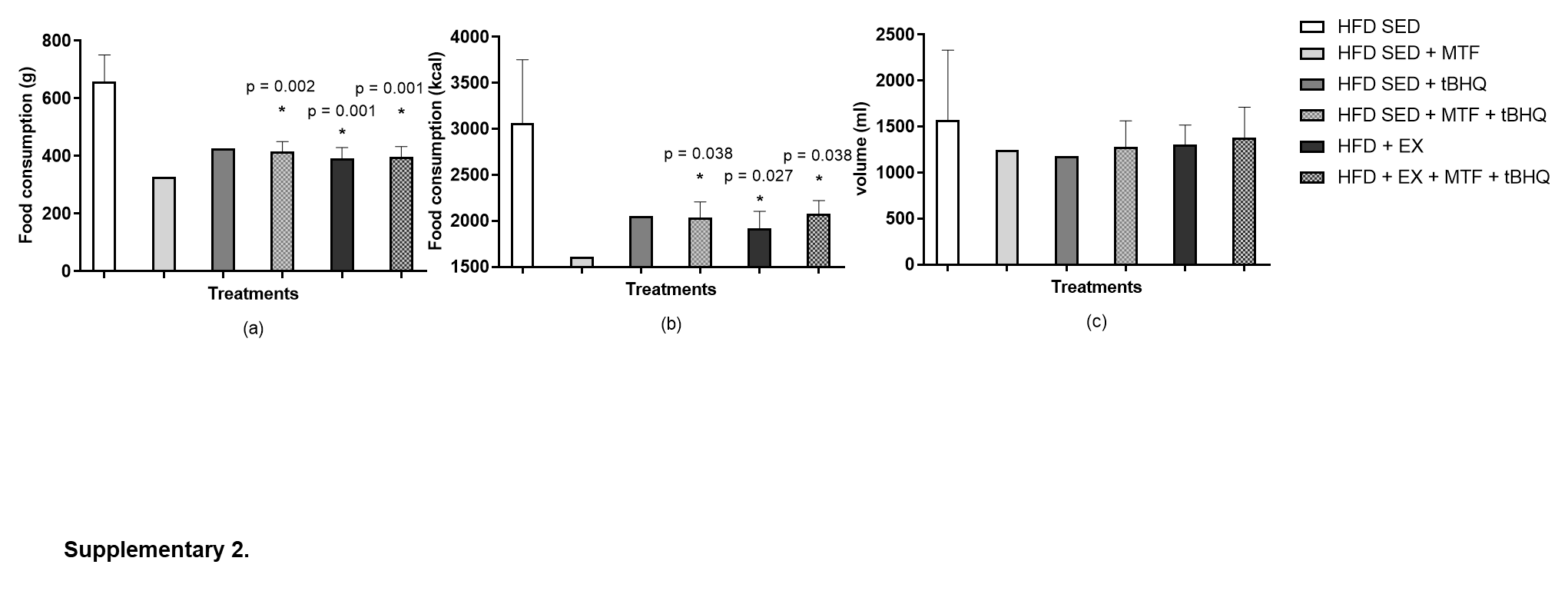
Supplementary figure 2. Food and water consumption of HFD-treated groups.**

Food consumption expressed in g (**a**) or kcal (**b**), and water consumption (**c**), were determined in HFD-rats and the respective treatments metformin (MTF), tBHQ and exercise (EX) and their combinations. The significant statistical differences between groups with respect to the HFD were marked with *. The exact probability value is indicated in the graph (The comparisons were established using ANOVA and a post hoc Holm-Sidak HFD n= 8, HFD + MTF n=5, HFD + tBHQ n=4, HFD + MTF + tBHQ n=11, HFD + EX n=11, HFD + EX + MTF + tBHQ n=8. * p < 0.05)
